# Skull bone tumor: a review of clinicopathological and neuroimaging characteristics of 426 cases at a single center

**DOI:** 10.1186/s40880-019-0353-0

**Published:** 2019-03-08

**Authors:** Hailong Liu, Xueying Zhang, Mingshan Zhang, Junping Zhang, Weihai Ning, Angela Yue, Rugang Zhao, Youliang Sun, Chunjiang Yu

**Affiliations:** 1 Department of Neurosurgery, Sanbo Brain Hospital, Capital Medical University, No. 50 Yikesong Road, Xiangshan Ave, Beijing, 100093 P. R. China; 2 Department of Neurosurgery, Chinese People’s Liberation Army General Hospital, Beijing, 100853 P. R. China; 3 School of Public Health, Peking University, Beijing, 100191 P. R. China; 4 Tate Cancer Center, Baltimore Washington Medical Center, University of Maryland, Glen Burnie, MD 21061 USA; 5 Department of Orthopedics, Beijing Ditan Hospital, Capital Medical University, Beijing, 10015 P. R. China; 6 School of Basic Medical Science, Capital Medical University, Beijing, 10069 P. R. China

Dear Editor,

Among the wide array of human neoplasms, bone and soft tissue tumors originating from the skull bone are extremely rare, making up < 2% of all the musculoskeletal tumors [1]. Skull bone tumors exist as a distinct entity because they include multiple subtypes, have complex regional anatomical structure, and require interdisciplinary therapy. According to the 2002 world health organization (WHO) histological typing [2], skull bone tumors can be divided into benign, malignant, and undefined neoplastic nature tumors (UNNTs). Given the lack of literatures systematically reporting this uncommon disorder and the small-scale sample size published studies on these tumor entities [3, 4], a deeper understanding of the clinicopathological features across skull bone tumor is necessary. We, therefore, conducted this retrospective analysis on bony tumor spectrum involving the primary and secondary lesions arising from the skull to document the various types of tumors encountered, and to analyze their epidemiological characteristics, clinicopathogical features and neuroradiographic parameters.

A total of 426 skull bone tumors including 27 subtypes diagnosed over a period of 10 years (from March 2005 to December 2016) were retrieved from the Sanbo Brain Hospital of the Capital Medical University (Beijing, China). The mean age of the investigated patients was 33.0 years (range, 4–81 years). Among all cases, 60 (14.1%) were diagnosed as benign tumors, 299 (70.2%) were malignant tumors and 67 (15.7%) were UNNTs (Additional file 1: Table S1). Skull metastatic tumors occupied only a small percentage of this cohort (24 cases, 5.6%). The most frequent malignancy was chordoma (175 cases, 41.1%), which was the most common observed tumor types among all cases. The most common benign tumors and UNNTs were osteoma (13 cases, 3.1%) and fibrous dysplasia (FD; 35 cases, 8.2%), respectively. There was a male preponderance (228 males and 198 females). Majority of patients were adults (345 cases, 81.0%), which were about four times of pediatric patients (81 cases, 19.0%). As shown in Fig. 1a, the incidence rate of all the cases increased rapidly until the age 40 years and the maximum incidence was in 31–40 age group accounted for 25.6% of the total cases. Figure 1b showed that the maximum incidences of the patients with benign tumor, malignant tumor or UNNT were all in 31–40 age group. Similar to the incidence rate, the mortality rate was substantially higher in males than in females. The age distribution of mortality had two peaks, one was between 11 and 30 years (11–20 years in male and 21–30 years in female), and the other was between 61 and 70 years (Fig. 1c).

**Fig. 1:**
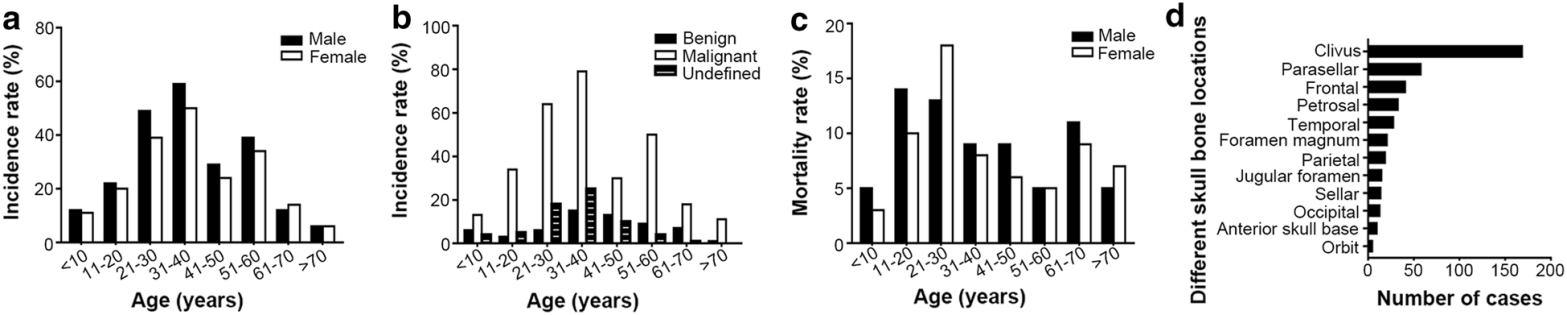
Distributions of epidemiological characteristics of 426 patients with skull bone tumors. **a** Distribution of incidence rate by age and gender. **b** Distribution of incidence rate by age and neoplastic nature (benign, malignant, and undefined neoplastic nature tumors). **c** Distribution of mortality rate by age and gender. **d** Summary of incidence of skull bone lesions presented in different locations

In regards to the clinicopathological characteristics, we found that the most common location was the clivus (169 cases, 39.7%), followed by parasellar (58 cases, 13.6%) and frontal (41 cases, 9.6%) regions (Fig. 1d). The parasellar region was more frequently associated with benign tumors (17/60 cases, 28.3%), while malignant tumors were found preferentially in the clivus (142/299 cases, 47.5%) and UNNTs tended to occur in the frontal bone (10/67 cases, 14.9%; Additional file 1: Table S1). The preoperative/pathological diagnostic rate (PPDR) was 41.7% in the benign group, 69.9% in the malignant group, and 71.6% in the UNNT group (Additional file 1: Table S1). After decades of development, multidisciplinary treatment consisting of surgical resection [gross-total resection (GTR) or subtotal resection (STR)], radiotherapy, chemotherapy and comprehensive therapy has been achieved for previously inaccessible disorders [5]. In the benign group, GTR was performed in all the patients with osteoma but in only 1/5 of the patients with myxoma because of the local invasion (Additional file 1: Table S1). The fronto-temporal approach was the most common approach for resecting tumors located in the parasellar region (25/58 cases, 43.1%), while the temporo-occipital approach was conducted most commonly in the malignant group (76/299 cases, 25.4%). Furthermore, radiotherapy and chemotherapy were recommended to the patients with malignant tumors by a proportion of 71.6% and 41.1%, respectively. Among the overall 426 investigated patients, local recurrence was observed in 146 (34.3%) patients after initial surgery during a median follow-up period of 56.0 months (range, 2–141 months). Patients with malignant tumors were the leading group for having recurrence, accounting for nearly 1/3 of the patients with recurrence. Additionally, 137 patients (40.6%, 71 males and 66 females) died during the follow-up period. It was noticeable that the survival rate of patients with myxoma (60.0%) and Langhans cell histiocytosis (75.0%) was lower when compared to that of patients with other non-malignant tumors (Additional file 1: Table S1).

To investigate the radiographic and histopathological characteristics, we analyzed the imaging phenotype of computed tomography/magnetic resonance imaging (CT/MRI) and pathological phenotype of tumor tissues. As shown in Additional file 2: Table S2, half of the benign tumors were observed to destroy the surrounding bones. Moreover, there was a larger proportion of benign tumors to have calcification as compared with malignant tumors and UNNTs. Multiple radiographic signs, such as the actinomorphous-shape destruction of haemangioma (Additional file 3: Figure S1A), the eggshell-like calcification of giant cell tumor (GCT, Additional file 4: Figure S2A, B) and the irregular structural calcification of chondrogenic tumors (Additional file 3: Figure S1B), provided satisfactory indication for accurate diagnosis of the benign tumors. Lateral locations were more frequently associated with benign tumors (81.7%) and UNNTs (67.2%), while malignant tumors were most frequently located at the midline (57.5%; Additional file 2: Table S2). Heterogeneous pattern of enhancement was chiefly observed in myxoma (5/5 cases, 100%), GCT (5/7 cases, 71.4%; Additional file 4: Figure S2C–E) and osteoblastoma (3/8 cases, 37.5%). Additionally, since severe erosion in the intraosseous meningioma was observed (Additional file 3: Figure S1C), the intraosseous meningioma was considered to be a benign tumor with malignant behavior. Furthermore, fluid–fluid levels (FFLs) could be considered as the differential diagnosis for confirming haemangioma since 5/6 (83.3%) haemangioma have alterations in FFL (Additional file 3: Figure S1D). MRI scans indicated that most of the benign tumors had clear boundaries (76.7%; Additional file 2: Table S2), which are consistent with the biological characteristics of benign tumors. Large cystic areas generally emerged in the myxoma (5/5 cases, 100%; Additional file 3: Figure S1E), vascular masses (14/16 cases, 87.5%) and chondrogenic tumors (56/79 cases, 70.9%), but not in hematopoietic tumors (1/18 case, 5.6%). Finally, based on the statistical analysis of diffuse weight image (DWI), we found that the higher diffusivity mainly demonstrated in malignant lesions (202/299 cases, 67.6%; Additional file 2: Table S2 and Additional file 3: Figure S1F). Pathological analysis revealed that GCT, a benign but locally aggressive tumor, was characterized by mounts of osteoclast-like giant cells with a spindle-shaped mononucleus (Additional file 4: Figure S2F). Positive staining for Phosphoglucomutase 1 (CD68) and Cytokeratin (CK14) was a typical feature of this tumor, suggesting its origin to be from osteoclasts (Additional file 4: Figure S2G, H). However, there is a lack of specific immunostaining index for the diagnosis of skull bone tumors [6]. Thus, it is necessary to establish diagnostic tools in routine pathological practice through the systematic identification of microscopic features.

Compared with the benign population of this study, it was worthy to note that the calcification as one benign index was found to be predominated in the chondrosarcoma because most of the chondrosarcoma in this study (49/57 cases, 86.0%) were at low grades with well differentiation. In addition, some specific neuroimaging signs such as the osteolytic lesion of solitary plasmacytoma of bone (SPB) and Ewing sarcoma (EWS, Additional file 5: Figure S3A), and the full clivus destruction of chordoma was observed. MRI was superior in identifying the invasive status to the surrounding tissues [7]. The invasion condition could be substantially divided into two subtypes based on the expansive (Additional file 3: Figure S1G and Additional file 5: Figure S3B, C) and invasive (Additional file 3: Figure S1H) pattern of MRI presentations. Besides, homogeneous enhancement was chiefly observed in SPB (6/9 cases, 66.7%), osteosarcoma (7/8 cases, 87.5%) and EWS (11/12 cases, 91.7%). Similar to the benign tumors, primary malignancies also originated from various tissues. Histologically, EWS was composed of numerous small round cells with uniform nuclei and eosinophilic cytoplasm, bordered indistinctly to each other (Additional file 5: Figure S3D) and it was diffusely positive for Microneme protein 2 (MIC2, CD99) and Friend leukemia integration 1 (FLI-1, Additional file 5: Figure S3E, F).

Aggressive bone destruction of skull tumor generally indicates a malignant process. In contrast, our findings showed that bone destruction was found in approximately 2/5 of UNNTs and mainly occured in the lateral skull bones (Additional file 2: Table S2). Some specific signs were obtained based on CT scans including the cystic destruction of aneurysmal bone cyst (ABC), the eggshell-like calcification of epidermoid cyst and the bone-forming calcification of FD (Additional file 6: Figure S4A). In the analysis of MRI features, we detected that more than 2/5 UNNTs cases didn’t show the clarity of frontiers with the invasive potential as named “undefined”, especially for FD (Additional file 2: Table S2, Additional file 6: Figure S4B, C). In the present study, FD (35 cases, 8.2%) represented the most frequent subgroup with different proportions of woven osseous tissues and bland fibroblasts (Additional file 6: Figure S4D). Collectively, conventional estimates concerning the neuroradiology and histopathology still remained the cornerstone in establishing a differential diagnosis [8].

Chordoma and chondrosarcoma (232/426 cases, 54.5%) were the most common lesions in this study. They have many similarities in clinicopathology and neuroradiology [9, 10]. Clinically, the conventional subgroup (131 cases, 30.8%) showed the highest prevalence among all the chordoma and the most common subtype of chondrosarcoma was myxoid chondrosarcoma (48/57 cases, 84.2%; Additional file 7: Table S3). We found that dedifferentiated chordomas mainly occurred in young patients (6/9 cases, 66.7%) and were mainly located in lateral skull base (4/9 cases, 44.4%). The dedifferentiated chondrosarcomas were more likely to occur in the midline region (4/5 cases, 80.0%). According to the MRI features, the homogeneity of T2 weight image (T2WI) supported the diagnosis of dedifferentiated masses. All the chordomas was observed to be able to destroy the skull base and their calcification index was decreased in the dedifferentiated group indicating a high degree of malignancy. In terms of treatment, the proportion of GTR was lower in the dedifferentiated group of chordoma and chondrosarcoma (33.3% and 20.0%, respectively) as compared with the other groups, while the proportion of patients receiving radiotherapy and chemotherapy was higher (100% and 80.0%, respectively; Additional file 7: Table S3). Statistical analyses of the expression of specific markers was summarized in Additional file 8: Table S4, which indicated that the positivity for podoplanin and Lysozye (Lys), and the negativity for ethylene/methacrylic acid (EMA), cytokeratin (CK) and cytokine 8/18 (CK8/18) were helpful in distinguishing chondrosarcoma from chordoma (Additional file 9: Figure S5). We further analyzed the overall survival by utilizing the Kaplan–Meier method. The results suggested that the patients with dedifferentiated chordoma had poor prognosis compared with the conventional and chondroid subgroups (*P* = 0.0005; Additional file 10: Figure S6A), however, there was no statistically significant difference in overall survival among the myxoid, mesenchymal and dedifferentiated subgroups of patients with chondrosarcoma (*P* = 0.069; Additional file 10: Figure S6B). Consequently, the documentation of observed clinicopathological and radiographic characteristics of chordoma and chondrosarcoma is important to aid clinicians’ better diagnosis these tumors in a systematic manner.

This study reported the largest series of skull bone tumors and provided a comprehensive assessment of their epidemiology, clinicopathology and neuroradiology to improve their differential diagnosis. The malignant tumors present the main subgroup across the whole neoplasms arising from skull bone and the operation combined with radiation remains the chief therapeutic strategy for the malignancies. It is noticeable that neuroradiography plays a critical role in approaching the differential diagnosis of skull bone tumors, thus providing an objective protocol for patient management.

Detailed materials and methods are available in the Additional file 11.

## Additional files

**Additional file 1: Table S1.** Summary of general information, clinicopathologic features and follow-up results for 426 patients with skull bone tumors.

**Additional file 2: Table S2.** Statistical analysis of CT and MRI characteristics of 426 cases with skull bone tumors.

**Additional file 3: Figure S1.** Representative CT and MRI features in the current study (A) Sagittal bone-window CT presenting the actinomorphous-shape destruction of haemangioma. (B) Axial CT showing the irregular structural calcification in chondrosarcoma. (C) Axial enhanced T1WI displaying the extensive erosion in intraosseous meningioma. (D) Axial T2WI showing the cystic space with FFLs in haemangioma. (E) Axial contrasted T1WI showing the large cystic areas in myxoma. (F) DWI scan showing the obvious hyperintensity in myxoma. (G) Axial Flair image showing the expansive involvement in EWS. (H) Coronary T1WI with enhancement showing the invasive involvement in haemingioperithelioma. T1WI, T1 weighted image; T2WI, T2 weight image; DWI, diffuse weight image; FFLs, fluid-fluid levels.

**Additional file 4: Figure S2.** Neuroimaging and histopathological findings in representative case of GCT. A 22-year-old man presented with mild frontal headache for about 3 months and the progressive blurred version for 1 month. Preoperative sagittal (A) and axial bone window (B) CT scans showing a large tumor in the sellar region with the hyperintensity and eggshell-like calcification. Preoperative axial T2WI (C) and DWI (D) displaying a solid-cystic lesion with hyperintensity on T2WI and hypointensity on DWI. Preoperative sagittal gadolinium-enhanced MRI (E) showing the lesion with heterogeneous enhancement. GCT consisted of mounts of osteoclast-like giant cells containing numerous round or spindle-shaped nuclei (F) and it was positive for CD68 (G) and CK14 (H). GCT, giant cell tumor; T2WI, T2 weight image; DWI, diffuse weight image. Scale bar, 10 μm.

**Additional file 5: Figure S3.** Neuroimaging and histopathological findings in representative case of EWS. A 48-year-old woman suffering from the relapsed EWS presented with a right parietal headache and left upper limb dyskinesia for approximately 3 months. Axial bone window CT scan (A) showing the primary EWS eroding the right parietal skull bone. Sagittal enhanced T1WI (B) noting the secondary tumor involving the normal cerebral tissues with obvious peritumoral edema. Intraoperative photograph (C) presenting the EWS metastasizing to the brain with expensive involvement. EWS constituted the uniform small round cells with round nuclei and clear eosinophilic cytoplasm (D) and it was immunopositive for CD99 (E) and FLI-1 (F). EWS, Ewing sarcoma; T1WI, T1 weighted image. Scale bar, 5 μm.

**Additional file 6: Figure S4.** Representative neuroradiological and histopathological images of FD A 25-year-old young woman suffered from frontal protrusion and facial appearance deforming for about 8 years. The preoperative coronary CT scan (A) showing the bone-forming calcification in the frontal bone. Preoperative T2WI (B) and contrasted T1WI (C) displaying the hypointensity and obvious enhancement with the ambiguous boundary in FD. The H.E. staining (D) showing the distributed bland fibroblastic cells and irregular trabeculae of woven bone. T1WI, T1 weighted image; T2WI, T2 weight image. Scale bar, 5 μm.

**Additional file 7: Table S3.** Summary of general information, clinicopathologic features and follow-up results for 232 patients with chordomas and chondrosarcomas.

**Additional file 8: Table S4.** Statistical analysis of immunostaining status of 232 patients with chordomas or chondrosarcomas.

**Additional file 9: Figure S5.** Representative immunostaining features of chordoma and chondrosarcoma (A–C) Micrographs showing that chordoma was immunopositive for EMA and CK and negative for D2-40. (D–F) Images showing that chondrosarcoma was immunonegative for EMA and CK and positive for D2-40. Scale bar, 10 μm.

**Additional file 10: Figure S6.** Survival analysis by Kaplan–Meier estimate among the patients with chordoma and chondrosarcoma (A) The survival of patients with dedifferentiated chordoma was poorer than the other groups (*P *= 0.0005). (B) There existed no significant difference of survival time among the three subgroups of patients with chondrosarcoma (*P *= 0.069).

**Additional file 11.** Additional methods and materials.

## Abbreviations

WHO: World Health Organization
UNNT: undefined neoplastic nature tumor
FD: fibrous dysplasia
SPB: solitary plasmacytoma of bone
PPDR: preoperative/pathological diagnostic rate
DWI: diffuse weight image
GTR: gross-total resection
STR: subtotal resection
GCT: giant cell tumor
FFLs: fluid–fluid levels
EWS: Ewing sarcoma
ABC: aneurysmal bone cyst
T2WI: T2 weight image
T1WI: T1 weighted image
EMA: ethylene/methacrylic acid
CK: cytokeratin
CK8/18: cytokine 8/18
