# Supplementary figures and images for: Skull bone tumor: a review of clinicopathological and neuroimaging characteristics of 426 cases at a single center
Source: Cancer Commun (Lond). 2019 Mar 8;39:8. doi: 10.1186/s40880-019-0353-0 (PMC6407197; doi:10.1186/s40880-019-0353-0)

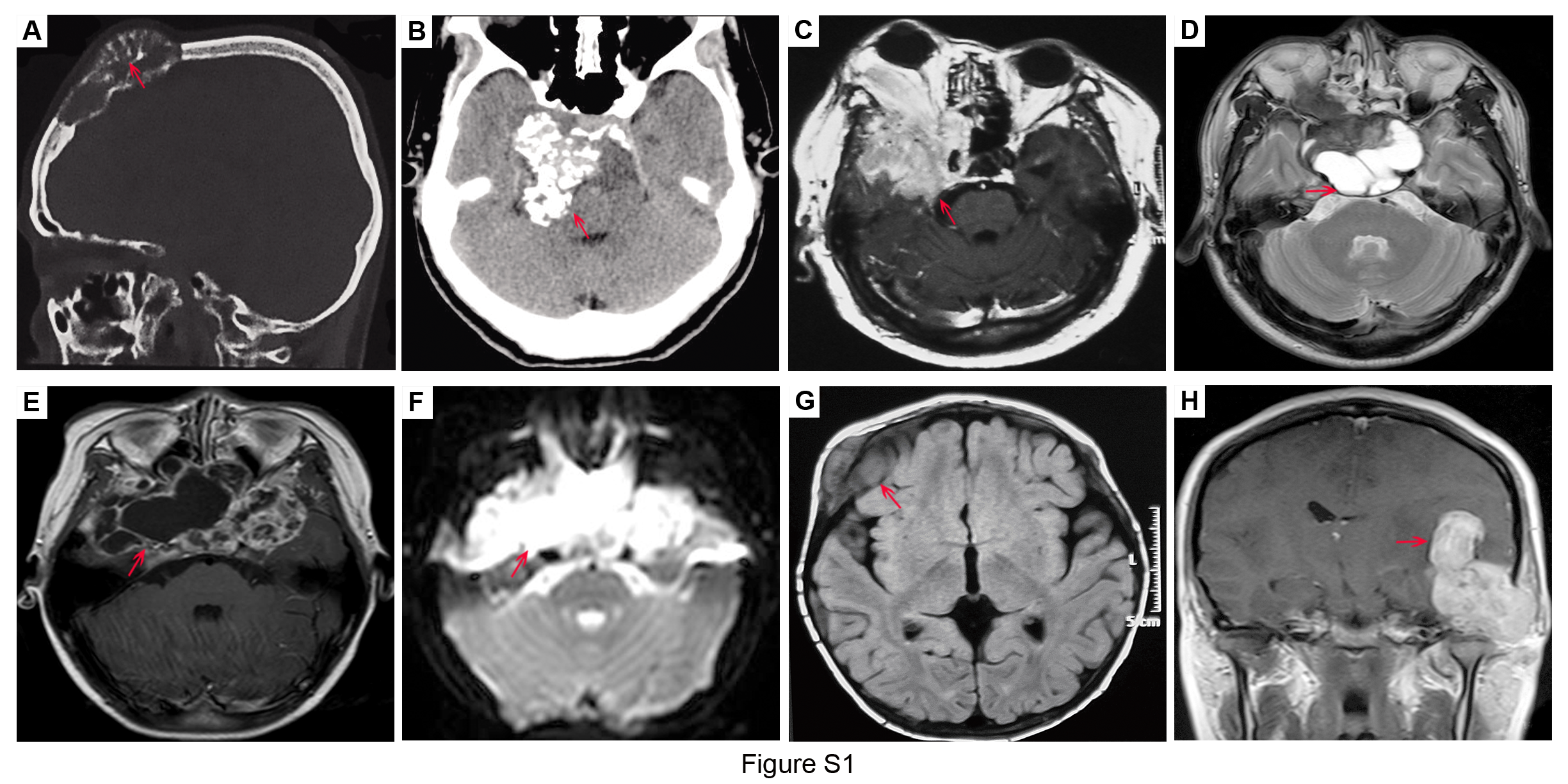

Supplement: Supplementary file 3 — Additional file 3: Figure S1. Representative CT and MRI features in the current study (A) Sagittal bone-window CT presenting the actinomorphous-shape destruction of haemangioma. (B) Axial CT showing the irregular structural calcification in chondrosarcoma. (C) Axial enhanced T1WI displaying the extensive erosion in intraosseous meningioma. (D) Axial T2WI showing the cystic space with FFLs in haemangioma. (E) Axial contrasted T1WI showing the large cystic areas in myxoma. (F) DWI scan showing the obvious hyperintensity in myxoma. (G) Axial Flair image showing the expansive involvement in EWS. (H) Coronary T1WI with enhancement showing the invasive involvement in haemingioperithelioma. T1WI, T1 weighted image; T2WI, T2 weight image; DWI, diffuse weight image; FFLs, fluid-fluid levels. [file 40880_2019_353_MOESM3_ESM.tif]

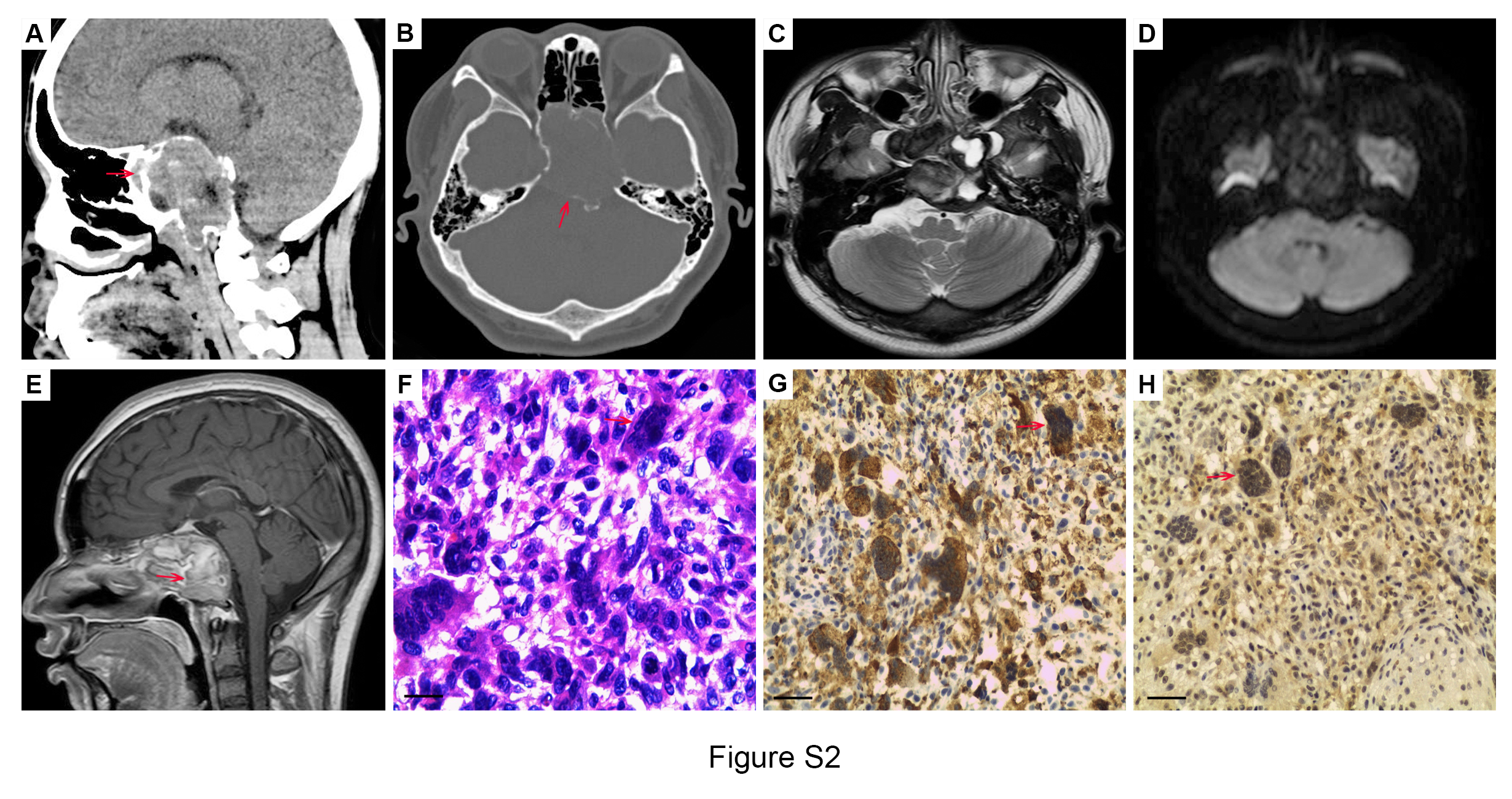

Supplement: Supplementary file 4 — Additional file 4: Figure S2. Neuroimaging and histopathological findings in representative case of GCT. A 22-year-old man presented with mild frontal headache for about 3 months and the progressive blurred version for 1 month. Preoperative sagittal (A) and axial bone window (B) CT scans showing a large tumor in the sellar region with the hyperintensity and eggshell-like calcification. Preoperative axial T2WI (C) and DWI (D) displaying a solid-cystic lesion with hyperintensity on T2WI and hypointensity on DWI. Preoperative sagittal gadolinium-enhanced MRI (E) showing the lesion with heterogeneous enhancement. GCT consisted of mounts of osteoclast-like giant cells containing numerous round or spindle-shaped nuclei (F) and it was positive for CD68 (G) and CK14 (H). GCT, giant cell tumor; T2WI, T2 weight image; DWI, diffuse weight image. Scale bar, 10 μm. [file 40880_2019_353_MOESM4_ESM.tif]

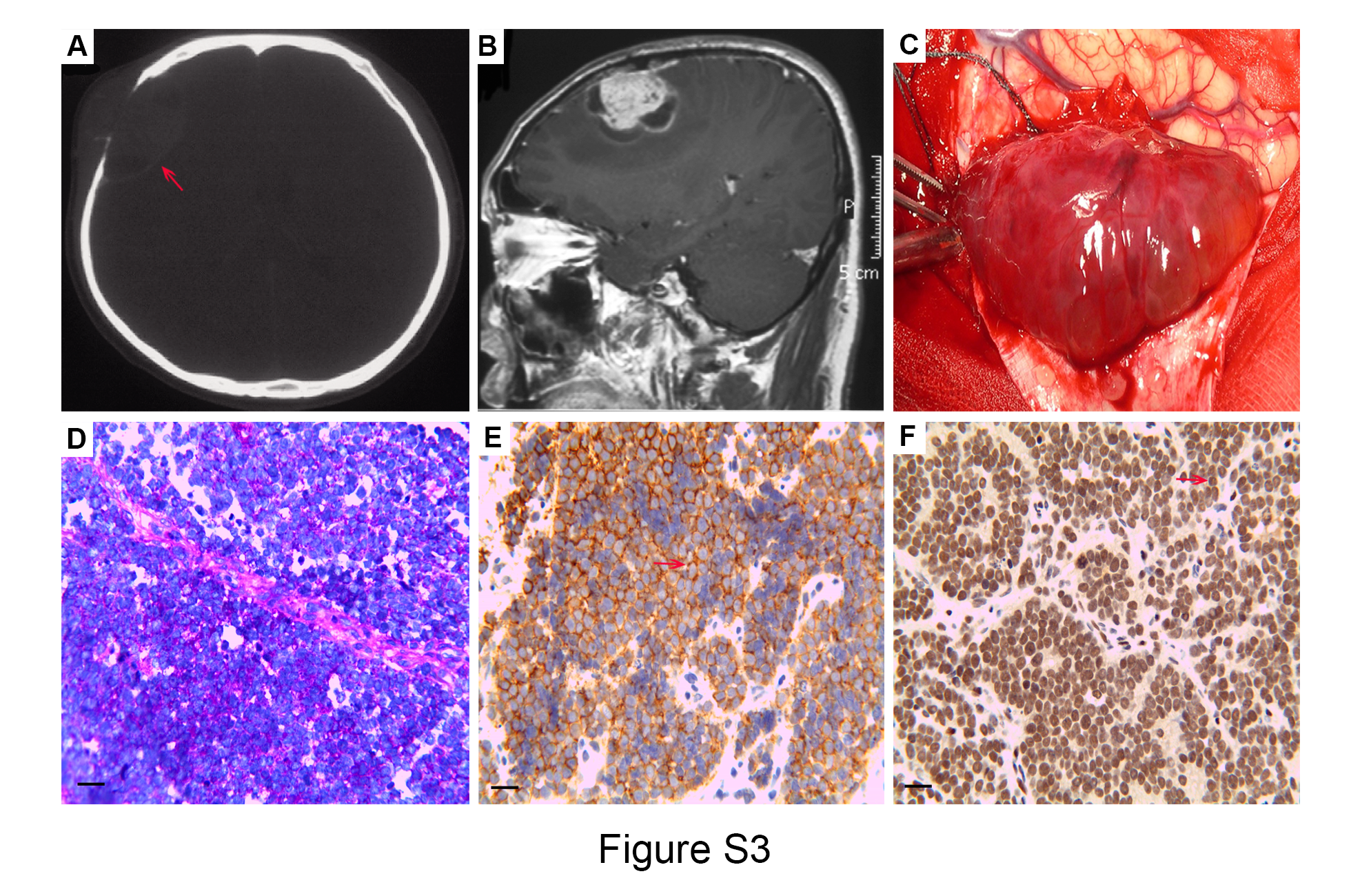

Supplement: Supplementary file 5 — Additional file 5: Figure S3. Neuroimaging and histopathological findings in representative case of EWS. A 48-year-old woman suffering from the relapsed EWS presented with a right parietal headache and left upper limb dyskinesia for approximately 3 months. Axial bone window CT scan (A) showing the primary EWS eroding the right parietal skull bone. Sagittal enhanced T1WI (B) noting the secondary tumor involving the normal cerebral tissues with obvious peritumoral edema. Intraoperative photograph (C) presenting the EWS metastasizing to the brain with expensive involvement. EWS constituted the uniform small round cells with round nuclei and clear eosinophilic cytoplasm (D) and it was immunopositive for CD99 (E) and FLI-1 (F). EWS, Ewing sarcoma; T1WI, T1 weighted image. Scale bar, 5 μm. [file 40880_2019_353_MOESM5_ESM.tif]

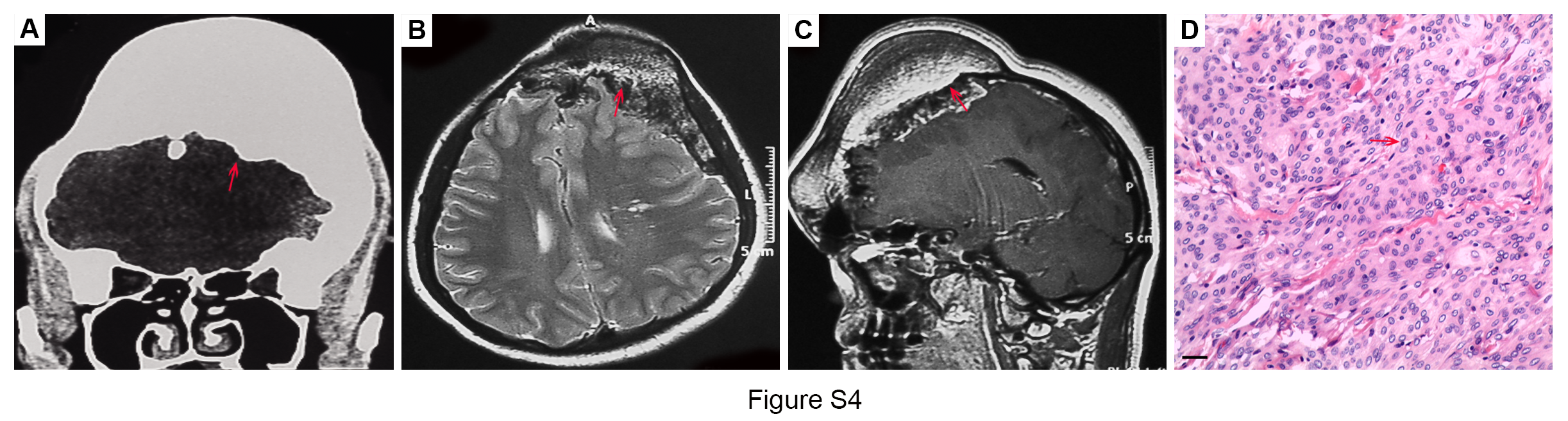

Supplement: Supplementary file 6 — Additional file 6: Figure S4. Representative neuroradiological and histopathological images of FD A 25-year-old young woman suffered from frontal protrusion and facial appearance deforming for about 8 years. The preoperative coronary CT scan (A) showing the bone-forming calcification in the frontal bone. Preoperative T2WI (B) and contrasted T1WI (C) displaying the hypointensity and obvious enhancement with the ambiguous boundary in FD. The H.E. staining (D) showing the distributed bland fibroblastic cells and irregular trabeculae of woven bone. T1WI, T1 weighted image; T2WI, T2 weight image. Scale bar, 5 μm. [file 40880_2019_353_MOESM6_ESM.tif]

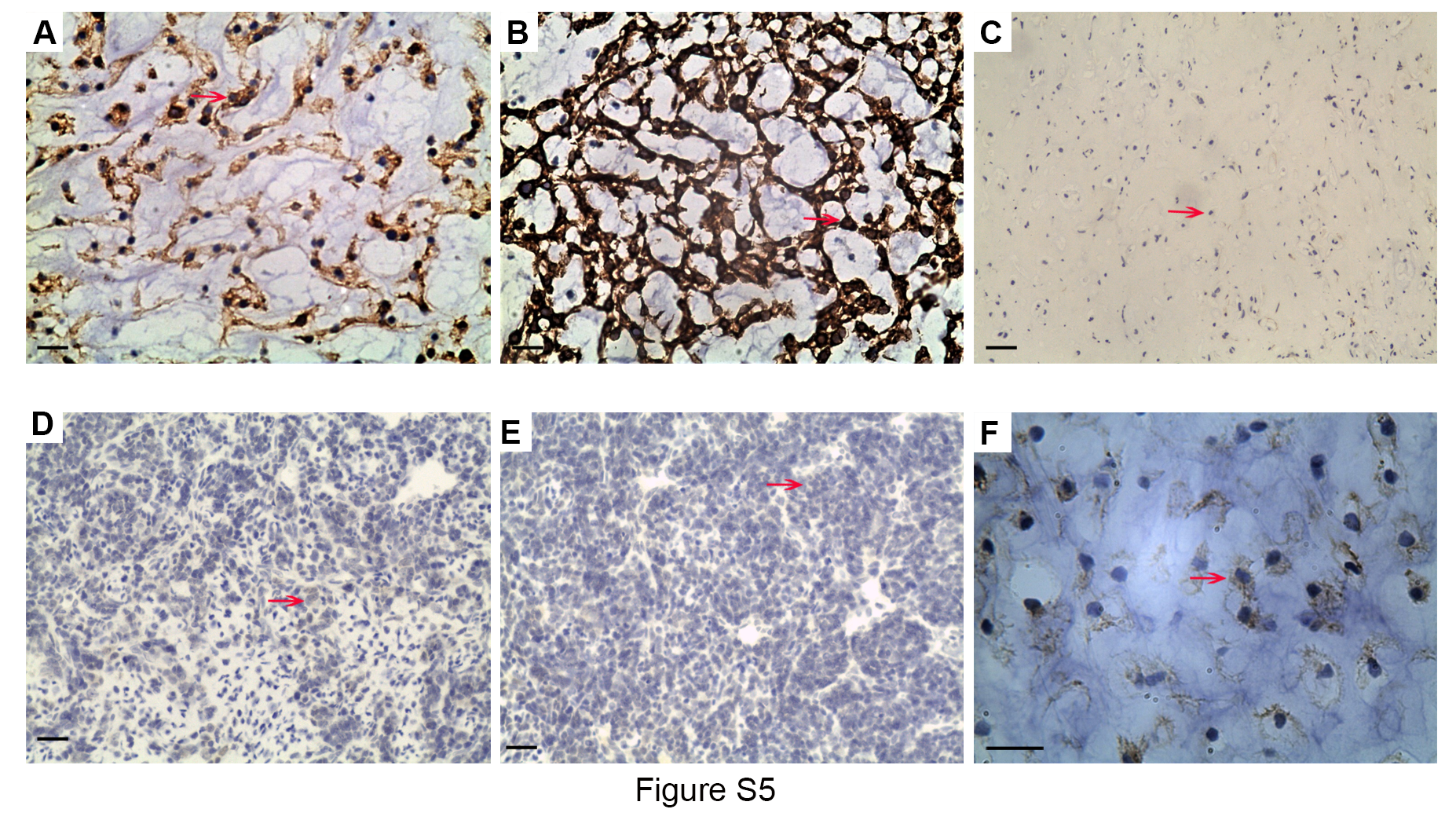

Supplement: Supplementary file 9 — Additional file 9: Figure S5. Representative immunostaining features of chordoma and chondrosarcoma (A–C) Micrographs showing that chordoma was immunopositive for EMA and CK and negative for D2-40. (D–F) Images showing that chondrosarcoma was immunonegative for EMA and CK and positive for D2-40. Scale bar, 10 μm. [file 40880_2019_353_MOESM9_ESM.tif]

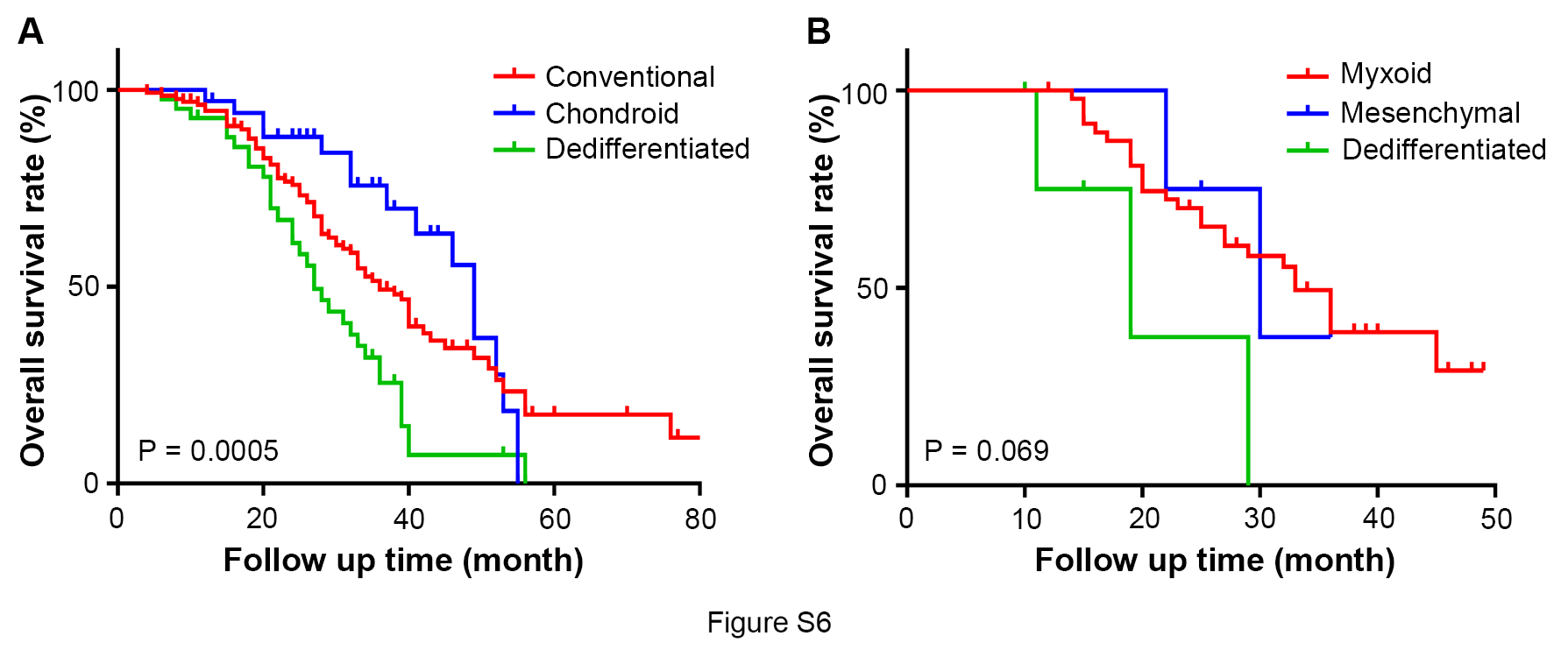

Supplement: Supplementary file 10 — Additional file 10: Figure S6. Survival analysis by Kaplan–Meier estimate among the patients with chordoma and chondrosarcoma (A) The survival of patients with dedifferentiated chordoma was poorer than the other groups (P = 0.0005). (B) There existed no significant difference of survival time among the three subgroups of patients with chondrosarcoma (P = 0.069). [file 40880_2019_353_MOESM10_ESM.tif]
