# Supplementary material for: Skull bone tumor: a review of clinicopathological and neuroimaging characteristics of 426 cases at a single center
Source: Cancer Commun (Lond). 2019 Mar 8;39:8. doi: 10.1186/s40880-019-0353-0 (PMC6407197; doi:10.1186/s40880-019-0353-0)
Supplement: Supplementary file 11 — Additional file 11. Additional methods and materials. [file 40880_2019_353_MOESM11_ESM.doc]

**Additional Methods and Materials**

**Study participates**

We identified 489 cases with skull lesions in our institution (from March 2005 to December 2016). Exclusion criteria were set up including the patients who didn’t receive operations or were lost for the follow-up and the definite soft tissue masses coming from the surrounding brain and meninges. So a total of 426 cases were included to the database. All enrolled patients were divided into three groups (benign, malignant and UNNT) including 27 subtypes.

The clinical characters including gender, age, location, PPDR, treatment, local recurrence and percentage of alive patients were detailed in Table S1. Initial examinations consisting of cranial CT and MRI scans were recommended to patients. The neuroimaging diagnosis confirmed independently by two neuroradiologists were retrospected and summarized in Table S2 giving attention to the following interests: CT features (bone destruction and calcification) and MRI presentations (midline/lateral location, boundary, soft tissue involvement, FFLs, cystic areas, enhancement, Flair and DWI features). The parameters (boundary and soft tissue invasion) were estimated based on the infiltration to the adjacent normal tissues on the contrasted T1WI. The FFLs and cystic areas were checked mainly according to the presentation on the T2WI. All samples were examined microscopically by two pathologists with the hematoxylin-eosin (H.E.) staining and immunohistochemical (IHC) staining. The preoperative impression was compared to the final histopathological diagnosis, defined as PPDR.

Patients underwent a routine follow-up at the outpatient or in the clinics at the 3-month interval for the first 6 months, then at the 6-month interval for the next 2 years and annually thereafter for 5 years. Patients suffering from possible local recurrence or metastases would increase follow-up frequency. MRI scans were performed to monitor the relapses. The prognosis associated materials were obtained through the outpatient records or telephone interviews.

We further reviewed the information of chordoma and chondrosarcoma with the aim in proposing an accurate preoperative diagnosis (Table S3). Based on the Chordoma Global Consensus Group (1, 2), chordoma comprised four subtypes: conventional, chondroid, dedifferentiated and clear cell subtype. Chondrosarcoma was categorized as myxoid (conventional), dedifferentiated and mesenchymal subtype. The neuroimaging characters and specific IHC markers (EMA, CK, CK8/18, D2-40 and Lys) were listed in Table S4. The number of positive staining cells was estimated by two pathologists counting 500 nuclei in 4 high-magnifying fields (400 ×) randomly as the percentage. Histological subtypes were also achieved by two pathologists in our institute.

**Statistical analysis**

Data statistical handling was performed using SPSS 19.0 and GraphPad Prism 7.0 software, and qualitative data were shown as cases (percentage) and quantitative data were presented as the median, range. The statistical analysis should be generated among the three major groups with meaningful results, but not correct by manipulating the subgroup data because of the rarity of some disorders. Comparison of mean values between multiple groups was evaluated by χ2 test. We estimated the numbers of occurrences and deaths among all the cases by applying the age course-specific incidence rate. The Pearson Chi-Square test was adopted for the comparison of values > 5 and the Fisher exact test was used for the values < 5. For all statistical methods, P < 0.05 was accepted as statistical significance.

**References**

1. Stachoitti S, Sommer J, Chordoma Global Consensus Group. Building a global consensus approach to chordoma: a position paper from the medical and patient community. Lancet Oncol. 2015;16(2):e71-83.
2. Bloch OG, Jian BJ, Yang I, et al. A systematic review of intracranial chondrosarcoma and survival. J Clin Neurosci. 2009;16(12):1547-51.
